# Powder Reuse Cycles in Electron Beam Powder Bed Fusion—Variation of Powder Characteristics

**DOI:** 10.3390/ma14164602

**Published:** 2021-08-16

**Authors:** Gitanjali Shanbhag, Mihaela Vlasea

**Affiliations:** Department of Mechanical & Mechatronics Engineering, University of Waterloo, Waterloo, ON N2L 3G1, Canada; gshanbhag@uwaterloo.ca

**Keywords:** electron beam powder bed fusion, powder reuse, Ti-6Al-4V, powder properties, density, flowability

## Abstract

A path to lowering the economic barrier associated with the high cost of metal additively manufactured components is to reduce the waste via powder reuse (powder cycled back into the process) and recycling (powder chemically, physically, or thermally processed to recover the original properties) strategies. In electron beam powder bed fusion, there is a possibility of reusing 95–98% of the powder that is not melted. However, there is a lack of systematic studies focusing on quantifying the variation of powder properties induced by number of reuse cycles. This work compares the influence of multiple reuse cycles, as well as powder blends created from reused powder, on various powder characteristics such as the morphology, size distribution, flow properties, packing properties, and chemical composition (oxygen and nitrogen content). It was found that there is an increase in measured response in powder size distribution, tapped density, Hausner ratio, Carr index, basic flow energy, specific energy, dynamic angle of repose, oxygen, and nitrogen content, while the bulk density remained largely unchanged.

## 1. Introduction

The performance of a powder-based additively manufactured part highly relies on the quality of the powder feedstock properties. Vock et al. [1] stated that the correlation between bulk powder behavior, powder layer organization, and final part quality is still not well understood. To improve the understanding of the powder–process–part relationship, there is a need to delve into quantifying the variability in powder properties, either as a function of print cycles, handling, or batch-to-batch variability, and investigate their effects on the resulting part properties. 

### 1.1. Powder Parameters of Interest for Electron Beam Powder Bed Fusion (EB-PBF) Processes 

When looking at powder feedstock for powder bed fusion (PBF) processes, Popov et al. [2] identified that some of the fundamental powder characteristics that need to be assessed include shape, particle size, composition, gas infusions, flowability, tendency to oxidize, and sintering/melting conditions. 

Particle morphology has a considerable influence on the powder bed packing density, and consequently on the final component density, where the more irregular the particles, the lower the final density [3]. Higher apparent densities are preferred, as they provide better heat conduction, reducing the risk of sample swelling and overheating [4] in the EB-PBF process. In terms of powder morphology, spherical or regular equiaxed particles, are less cohesive and tend to flow freely, arrange and pack more efficiently than irregular or angular particles [5]. As shape deviates from spherical, the interparticle friction increases and furthermore detrimentally affects the powder flowability and packing efficiency. Powder morphology can also affect the mechanical properties, microstructure, and surface finish of the final component. Consequently, Medina [6] emphasizes that powder morphology examination should be performed to identify particle shape, the presence of satellites, foreign particles, or contamination. Most powders for PBF processes are manufactured via atomization. Powders used in the EB-PBF process are typically manufactured via plasma atomization (PA). PA is the process of melting a wire spool feedstock of metal with a plasma torch and cooling it in an inert tower [7]. Powder particles manufactured by PA are usually spherical with minimal satellites and pores. 

Particle size distribution (PSD) is a significant parameter in determining the minimum layer thickness, the minimum achievable feature size in final parts, and affects the powder–energy source interaction. The EB-PBF process uses a nominal PSD between 45 μm and 105 μm. Simchi [8] explained that a deviation in the PSD can lead to in-situ powder segregation and layer streaking due to coarser particles being pushed away from the powder bed. This could lead to variations in build quality. 

The flowability of the powder is also highly important, to ensure uniform layers when dispensed, distributed, and/or spread onto the build area. It is generally understood that in order to obtain powder layers with homogeneous density, it is important to ensure that the powders are free-flowing and exhibit good flow properties. Powder flowability behavior can be correlated to the size, shape, moisture content, and packing efficiency of the powder particles [9]. For example, larger and spherical particles tend to flow better than smaller and irregular particles. The angle of repose (AOR) can be used to characterize the flowability of powders [1]. The AOR is affected by various cohesive forces: Van der Waals, electrostatic, and capillary, as well as the contact forces between powder particles. Teferra [10] states that powders that show a low AOR are categorized as non-cohesive, highly flowable powders and can be transported using gravitational force or extremely little energy. Powders with high AOR values are characteristic of cohesive powders and may lead to sporadic or intermittent flow. Powder rheometry characterization provides a suite of in-depth powder performance metrics such as tap density, apparent density, dynamic flow testing, dynamic angle of repose, shear index, and cohesiveness. 

Inert gas fusion analysis (i.e., LECO) provides quantitative data on the absolute oxygen and nitrogen content in the powders. This analysis is essential to understand whether the evolution of the oxygen and nitrogen content in the reused Ti-6Al-4V powder is within the allowable concentration limits defined by ASTM 2924-14 [11] (i.e., <0.20 wt.% for oxygen and <0.05 wt.% for nitrogen). 

It is of interest to quantify the effect of powder morphology, PSD, powder flowability, oxygen, and nitrogen content on the EB-PBF processes as a function of powder reuse.

### 1.2. Powder Parameter Studies in EB-PBF of Ti-6Al-4V

There has been an increasing interest in fabricating parts using EB-PBF. To continually improve the process, it is important to identify, address, and overcome some of the process limitations. Debroy et al. [12] have identified that the cost of a manufactured part in PBF processes is essentially contributed to by the additive manufacturing equipment, feedstock material, manufacturing, and indirect costs [13]. Specifically, when looking at the cost of one build via EB-PBF, Baumers et al. [14] have identified that the feedstock powder makes up to ~28% of the total cost of the build. Therefore, we can conclude that the cost of the EB-PBF process heavily relies on the reusability of powders and may not be cost-affordable for complex applications if the unmelted powder in the build bed is not reused. Thus, the evaluation of the maximum number of allowable powder reuse cycles is an essential factor to assess process affordability for a specific part or application. Reusing Ti-6Al-4V powder in EB-PBF can result in changes in the chemical composition, powder morphology, powder size distribution, and flowability, resulting in changes in the properties of the solidified material [15,16,17].

Powder morphology has been observed to be modified after reusing Ti-6Al-4V powders in EB-PBF. Tang et al. [18] observed that the particles became less spherical, had fractures, protrusions, and concave sites after reusing. They also observed an increase in surface roughness and distortions in the final part. Strondl et al. [5] observed irregularities, impact marks, satellites, and stacked particles in the recycled powder. Similar findings were reported by Mohammadhosseini et al. [19], where satellites and aggregation of particles in the reused powder were observed. 

Some studies also reported flowability results for virgin and reused powders. Tang et al. [18] observed that the reused powder showed lower flowability when measured by Hall flowmeter and attributed this to the blasting process, which led to irregular particle morphology and impact marks on the particle surface. Contradictory to this, Mohammadhosseini et al. [19] observed no change in the flowability, when measured by Hall flowmeter and Carney funnel, after reuse. 

Studies on the effect of reusing powder on chemical composition showed that reusing Ti-6Al-4V can lead to an increase in oxygen content. Sun et al. [20] observed that after 30 reuses, there was a 35% increase in oxygen content in the reused powder, from 0.15 wt.% in virgin powder to 0.20 wt.%. Petrovic and Niñerola [21] observed that the oxygen content exceeded the 0.20 wt.% limit after 12 reuse cycles, where the initial oxygen content in the virgin powder was 0.14 wt.%. They attributed this increase to the humidity pickup from the inner walls of the machine. Similarly, Tang et al. [18] observed that the oxygen content increased from 0.08 wt.% to 0.19 wt.% after 21 reuse cycles. They attributed this oxygen increase to exposure of powder to the air, including processing in the powder recovery system and sieving. An increase in oxygen content typically results in an increase in the strength but lowers the toughness and ductility of the final part [15]. Furthermore, Leung et al. [22] observed that the oxides in an oxidized powder may enable pore formation (and subsequently stabilize the pores) during the manufacturing process. Therefore, the mechanical properties of the parts will be varying with the number of reuse cycles. 

Based on these studies, a powder suitability criterion can be created for reused powders (see Table 1), for use in the EB-PBF process, to understand what type of powder performance metric behavior is suitable with respect to the morphology, size distribution, sphericity, basic flow energy, specific energy, density, Hausner ratio, Carr index, cohesive index, angle of repose, oxygen content, and nitrogen content.

EB-PBF machines store about 100–180 kg of powder (depending on the machine model) in the hoppers and it is often impossible to refill these hoppers with powder from the same reuse cycle. Hence blends of powders, either a mixture of virgin and reused or a mixture of different reuse cycles, are frequently used for manufacturing parts and such blend performances also need to be evaluated. These practices often pose challenges in isolating the effects of powder reuse in the above-mentioned studies. In addition, most published studies focus on assessing the effect of powder reuse on only a few specific powder performance metrics. Therefore, there is a need to assess the different powder characterization techniques and obtain the various powder performance metrics associated with these techniques. This needs to be done by precisely isolating the powder blends used in the build, as well as performing a comprehensive study on the effects of powder reuse on powder properties. Thus, the current comprehensive manuscript aims at advancing the authors’ preliminary study [23], by assessing different powder characterization techniques and obtaining the various powder performance metrics associated with these techniques to identify the key performance features of reused powder and powder blends. 

## 2. Materials and Methods

The Grade 5 plasma-atomized Ti-6Al-4V powder (Batch number: P1321, Advanced Powders and Coatings, Montreal, Quebec, Canada) was obtained in its pre-alloyed form with a size range of 45–105 μm. The chemical composition of the powder conforms to ASTM F2924 for a Grade 5 Ti-6Al-4V powder (according to batch information provided by the powder supplier). A total of seven powder types were assessed for this study. The powder obtained from the supplier is referred to as the virgin powder (henceforth known as Genesis 0 or G_0_). It should be noted that the G_0_ powder did not undergo any processing in the EB-PBF machine, blasting in the powder recovery system, or sieving to remove the fine powder particles. Therefore, this powder type may be considered an anomaly for the various powder characterization. Nevertheless, the results for G_0_ are presented in order to compare the results for all other powder types and use G_0_ as the baseline. Powder that was used once (Genesis 1 or G_1_), was passed through a vibrating sieve (mesh size −140 + 325, i.e., 44 µm to 105 µm) to recover the powder for reuse. Genesis 2 (G_2_) and Genesis 3 (G_3_) powders were similarly obtained after printing parts with G_1_ and G_2_ powder, respectively. For every powder type, approximately 500 g of powder was collected in a metal can. These cans were subsequently sealed and rolled/tumbled, on a jar-mill (Labmill 8000, Gardco, Pompano Beach, FL, USA), at 40 revolutions per minute (RPM), for 24 h in order to homogenize the sample before conducting any characterization. G_1_ and G_2_ powders were blended in equal wt.% to obtain the GB_12_ blend and compare its properties with the other powder types. Similarly, GB_13_ and GB_23_ blends were obtained based on equal wt.% of powder constituents. Table 2 summarizes the nomenclature and description of the various powder types investigated for the current study. The individual powder geneses were first collected in metal cans and rolled for 24 h, as mentioned earlier. These were then mixed in equal parts to create the respective blends. This blended powder was also collected in metal cans, sealed, and rolled for another 24 h to ensure homogeneity and proper mixing in the blend. Therefore, the blended powders see a total rolling/tumbling time of 48 h as compared to 24 h for the unblended powders. All printing was done on an Arcam A2X machine using the default parameters provided for Ti-6Al-4V (Theme 5.2.52, Arcam A2X, GE Additive, Gothenburg, Sweden). To ensure that a consistent beam scanning strategy was used for all builds, the parts built were constant across the 3 printing cycles. 

In order to assess the powder particle morphology, field emission microscopy (SEM, Zeiss Ultra and Tescan VEGA3, Munich, Germany and Pittsburgh, PA, USA, respectively) observations were performed. A Camsizer X2 (Retsch GmbH, Düsseldorf, Germany) was used to measure the PSD of the various powder types. Two grams of powder was used for each test performed on the Camsizer. The system uses the principle of digital image processing [24], where the dispersed particles pass in front of LED light sources and their shadows are captured with two digital cameras. The Retsch software (Version 6.7, Retsch GmbH, Düsseldorf, Germany) analyzes the size of each particle captured by the camera and calculates the respective distribution curves. 

**Figure 1:**
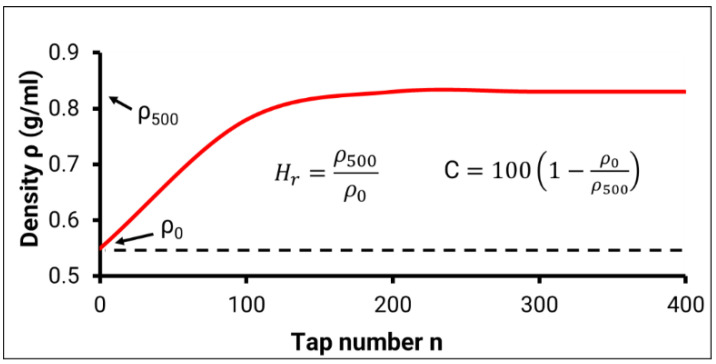
Example of compaction curve obtained by GranuPack—illustrating bulk density (*ρ*_0_), tap density (*ρ*_500_), Hausner ratio ($H_{r}$), and Carr index (C).

Powder rheology was investigated using a powder rheometer (FT4, Freeman Technology, Gloucestershire, UK), rotating drum (Granudrum; Granutools, Liège, Belgium) and an automated tap density instrument (GranuPack; Granutools, Liège, Belgium). Performance metrics were collected, such as the basic flow energy (BFE), specific energy (SE), bulk density (*ρ*_0_), tap density after 500 taps (*ρ*_500_), Hausner ratio ($H_{r}$), and Carr index (C). To characterize the resistance to flow, eleven test cycles were run with a condition cycle run between each test. A total of 25 g of powder was used for each test performed on the FT4. During the tests, the precision blade was rotated downwards and upwards through the fixed volume of powder to establish a flow pattern, where the resistance of the powder to the blade yielded the bulk flow properties. The BFE is defined as the energy required to displace a powder when the blade is moving downward, and the powder is constrained. As described by Freeman and Fu [25], the SE measures the powder’s flowability as the blade rotates upward and the powder is unconfined as there is no enclosure at the top of the vessel. The GranuPack measures the evolution of the powder density as a function of the tap number to obtain a compaction curve (as shown in Figure 1), which is used to calculate the $H_{r}$ and C values. 35 g of powder was used for each test performed on the GranuPack. $H_{r}$ is a number that is correlated to the flowability of a powder and is calculated using the formula $H_{r}=\rho_{500}/\rho_{0}$, where $\rho_{500}$ is the tapped density of the powder after 500 taps and $\rho_{0}$ is the initial bulk density of the powder. C is related to the compressibility of a powder and is calculated by the formula $100\;\left(\rho_{500}\;-\;\rho_{0}\right)/\rho_{500}$, where $\rho_{0}$ is the initial bulk density of the powder and $\rho_{500}$ is the final tapped density of the powder after 500 taps. 

The GranuDrum instrument (Granutools, Liège, Belgium) is used to determine the dynamic angle of repose and the cohesive index. The GranuDrum is composed of a horizontal drum half-filled with powder that rotates around its axis at an angular velocity ranging from 2 rpm to 60 rpm. A total of 50 g of powder was used for each test performed on the GranuDrum. In total 17 velocities were tested, from 2 rpm to 20 rpm at increments of 2 rpm followed by 25 rpm to 60 rpm at increments of 5 rpm. To minimize internal sequence effects, a different velocity sequence was used for each replicate (3 replicates in total) such that the sequences for all replicates are minimally correlated. A camera takes snapshots for each angular velocity and the software calculates the flowing angle of powder (or AOR) and the cohesive index (CI) values. 

Chemical analysis for the various powders were performed using inert gas fusion on a LECO TCH 600 (Leco Corporation, St. Joseph, MI, USA) instrument to analyze the oxygen and nitrogen content in the powders. 3 g of powder was used for each test performed on the LECO TCH 600. All powder characterisation experiments were performed in triplicate and the average values are reported in this manuscript. 

## 3. Results

As the number of reuse cycles increases, properties such as chemical composition, surface features (e.g., surface roughness and overall particle roundness), and physical and thermal properties are expected to change. Therefore, understanding the powder behavior with reuse is important for both cost and quality control. 

### 3.1. Observations of Changes in Powder Properties with Reuse Cycles

**Figure 2:**
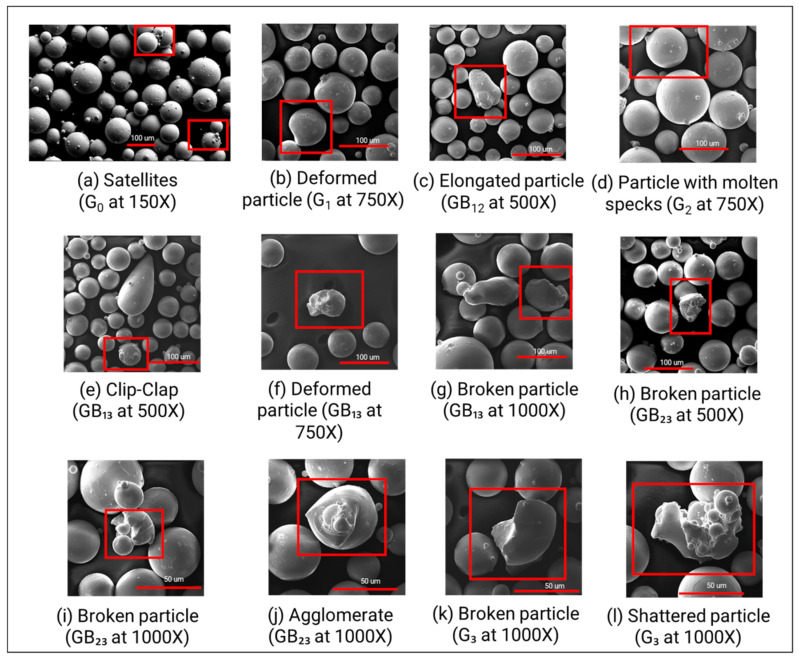
SEM micrographs for all powder types depict (**a**) satellites in G_0_ powder; (**b**,**f**) deformed particle in G_1_ and GB_13_ powders, respectively; (**c**) elongated particle in GB_12_ powder; (**d**) particle with molten specks in G_2_; (**e**) clip-clap in GB_13_ powder; (**g**,**h**,**i**,**k**) broken particles in GB_13_, GB_23_ and G_3_ powders; (**j**) agglomerate in GB_23_ powder and (**l**) shattered particle in G_3_ powder. Nomenclature used to describe these micrographs was first defined by Popov et al. [26]. A Zeiss Ultra SEM instrument was used to capture the image shown in (**a**); all other images were captured using the Tescan VEGA3 instrument.

The powder is predominantly spherical in its as-received (or virgin) condition. Figure 2 presents the high magnification SEM micrographs for all different powder types. These micrographs help define and depict defects such as satellites (Figure 2a), elongated particles (Figure 2c), broken particles (Figure 2g,h,i,k), deformed particles (Figure 2b,f), particle with molten specks (Figure 2d), clip-clap (Figure 2e), shattered particles (Figure 2l), and agglomerates (Figure 2j). The nomenclature and morphology of G_0,_ G_1_, G_2_ and GB_12_ powder types have been previously described in [23,26]. Other authors have also reported defects such as non-spherical particles and presence of agglomerates after reusing. Sun et al. [20] observed noticeable deformations and distortions on the surface of reused EB-PBF powder particles. Cordova et al. [27] observed that reused powder, in laser PBF processes, exhibits a deformation towards a teardrop shape and a rougher surface due to remelting. 

The deformed, broken, clip-clap, and shattered particle defects are attributed to the recovery via the blasting procedure. The homogenization of powder via tumbling on a jar-mill may result in numberless collisions between particles, and in friction and wear in the presence of air, and therefore the tumbling procedure may be another reason for these defects. The particles with molten specks and elongated particles are attributed to the temperature conditions that lead to overheating and smelting of the particles and satellites [26]. The agglomeration of powders is attributed to the high temperature of the process. Agglomerated particles result from the diffusion bonding obtained during preheating (to allow charge dissipation through the powder bed and reduce particle ejections resulting from the interaction of the electron beam during melting). The SEM micrographs (Figure 2) make it qualitatively evident that recovering and reusing the powder from the powder cake has changed the powder morphology significantly. 

**Figure 3:**
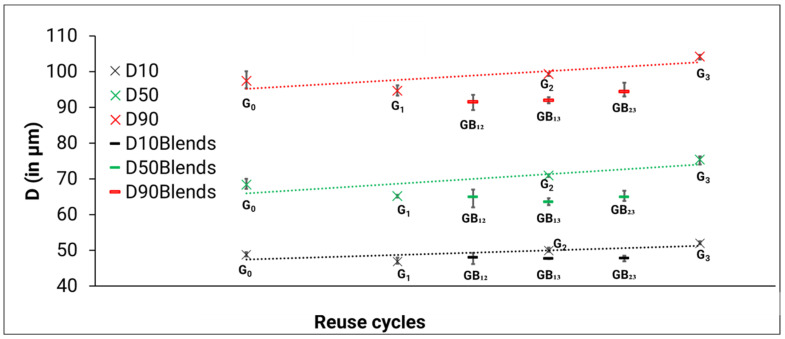
D_10_, D_50_ and D_90_ values; for all powder types. Error bars represent the min–max range.

Figure 3 shows the D_10_, D_50_, and D_90_ values of the different powders investigated. The D_50_ (median value), is described as the diameter where half of the population lies below this value. Similarly, 90% and 10% of the distribution lie below D_90_ and D_10_, respectively. It is worth noting that the G_0_ powder has not undergone any processing in the EB-PBF machine, nor blasting or recovery through the sieve. This contributes to the discrepancies below 44 µm in the PSD of G_0_ when compared to the other powder types. As expected, the D_10_, D_50_, and D_90_ values for GB_12_, GB_13_, and GB_23_ lie between their respective genesis powders. This is because the authors ensured that the powder blends were made from equal wt.% of powder constituents and mixed thoroughly before characterization. The D_10_, D_50_, and D_90_ values for the individual genesis powders (i.e., G_1_, G_2_, G_3_) show an increasing trend (Figure 3). In other words, one can say that the D_10_, D_50_, and D_90_ values increase with an increase in number of reuse cycles. Specifically, a 7%, 10%, and 7% increase was observed in the D_10_, D_50_, and D_90_ values, respectively, for G_3_ powder when compared to the G_0_ powder.

Slotwinski et al. [28] also reported an increase in particle size with increasing number of reuse cycles in laser powder bed fusion (LPBF). They associated this observation to the consolidation and loss of the small particles. Grainger [29] also noticed a disappearance of smaller particles with an increase in the number of powder reuse cycles in LPBF. Although the LPBF process does not use the same energy source, nor result in a powder cake after sintering, the powders are exposed to sputter and undergo a sieving process to recover the powders for reuse; similarities in trends with EB-PBF are observed in the present work.

**Figure 4:**
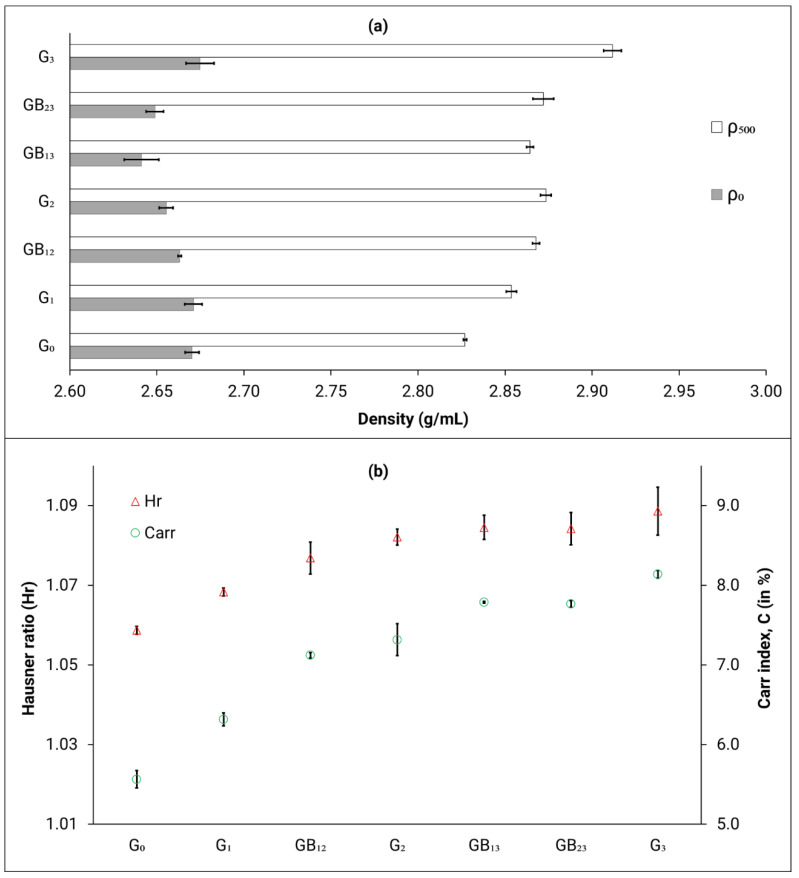
(**a**) *ρ*_0_ and *ρ*_500_ values (**b**) *Hr* and *C* values; for all powder types. Error bars represent the standard deviation.

The bulk density (*ρ*_0_) measured with the GranuPack instrument indicates that the values for the individual genesis powders and the powder blends do not significantly change with reuse (as seen in Figure 4a). Similar results were observed by Tang et al. [18], where the *ρ*_0_ remained unchanged after 21 reuse cycles. A powder with good flowability is usually characterized by a high *ρ*_0_ value. This is because free-flowing particles (with minimum interparticle adhesion) would be able to find an optimum arrangement and pack densely, therefore corresponding to a higher *ρ*_0_ value. For such a powder, the possibility of a density increase during tapping is limited, and therefore the ratio of tap density (*ρ*_500_) to *ρ*_0_ (also known as the Hausner ratio) would be close to unity.

The *ρ*_500_ values are presented in Figure 4a. It is observed that the tap density increases with the number of reuse cycles. Specifically, a 3% increase was observed in the *ρ*_500_ value for G_3_ powder when compared to the G_0_ powder. For the most part, the tap density values for the powder blends lie between their respective genesis powders. The difference between the bulk and tapped densities is significant and increases with an increase in number of reuse cycles. Interparticulate interactions are usually larger for poorly flowing powders and therefore a greater difference between the bulk and tapped densities is observed [27]. This suggests that the powder flowability is reduced when the number of reuse cycles increases.

The Carr index (C) is the measure of the extent to which a powder can be compressed (without deforming the particles). The compressibility of the powder is expected to affect the continuity and uniformity of the powder layer, with lower C values in favor of the formation of denser layers. As mentioned earlier, $H_{r}$ is an index that helps assess the flowability of the powder. According to Goyal et al. [30], for excellent compressibility and flowability, the C (%) and $H_{r}$ should be lower than 10% and 1.11, respectively. The $H_{r}$ and C values measured in this study, are presented in Figure 4b. When looking at all powder types, a good correlation is observed between the $H_{r}$ values and number of reuse cycles as well as Carr index and number of reuse cycles, such that an increase in these metrics is observed with an increase in number of reuse cycles. For the most part, the $H_{r}$ and C values for the powder blends lie between their respective genesis powders. All the values indicate that the flowability and compressibility is excellent (as defined by Goyal et al. [30]); however, the increasing trend suggests a degradation in the powder flowability characteristics. These plots strongly indicate that the powder has deteriorated from its virgin state. Specifically, a 3% and 30% increase was observed in the Hr and C values, respectively, for G_3_ powder when compared to the G_0_ powder. This observation is also supported by the general increase of PSD and changes in powder morphology observed as a function of increased reuse cycles.

This degradation of flow properties of the powder blends, as well as the reused individual genesis powders is attributed to the deviation in the powder morphology as observed in the SEM micrographs (Figure 2). Such deviations from the spherical morphology are expected to not only decrease flowability but may also lead to uneven layer formation during raking and ultimately may result in powder bed non-uniformity across the build bed, as mentioned in Table 1. On the other hand, the virgin (or G_0_) powder is observed to be more spherical and therefore flows easily due to lower surface friction and mechanical interlocking, thus displaying a lower value for $H_{r}$ and C metrics. The packing ability of powder particles influences the sintering of the powder layer [31]. As Neira-Arce [32] describes, uniform and homogeneous layers are crucial to ensure that there is proper heat conduction and for achieving dimensional accuracy, which in turn reduces the risk of swelling or overheating in EB-PBF parts.

**Figure 5:**
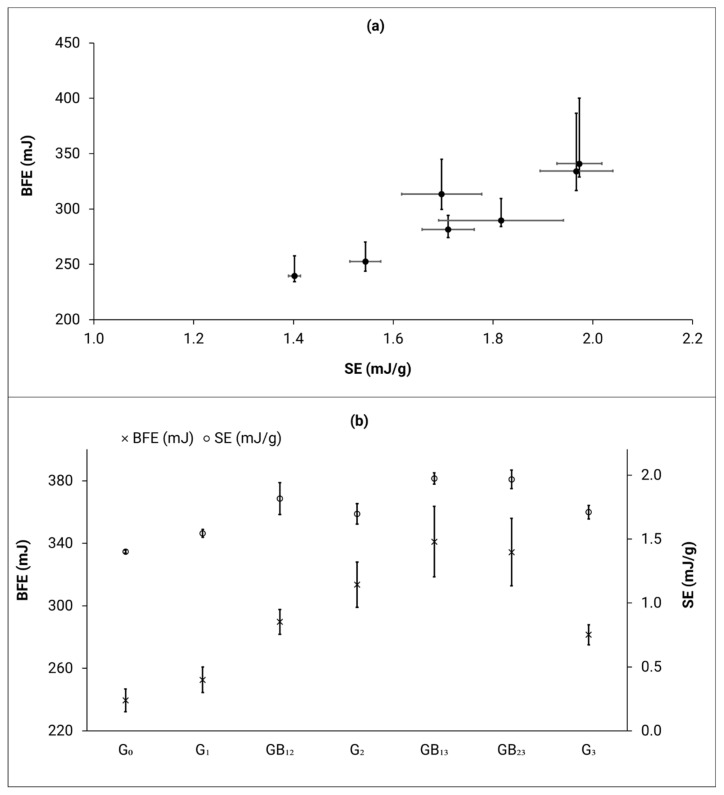
(**a**) BFE vs. SE, (**b**) BFE and SE values; for all powder types. Error bars represent the min–max range.

The BFE and SE (Figure 5b) values for the reused powders and blends have also increased with an increase in number of reuse cycles. Specifically, an 18% and 15%, increase was observed in the BFE and SE values, respectively, for G_3_ powder when compared to the G_0_ powder. The effect of larger BFE and smaller bulk density of the powder blends might result in a more uneven layer distribution. When looking at the individual genesis powders (Figure 5b), it can be concluded that both BFE and SE show an increase in values with an increase in number of reuse cycles, with G_3_ being an exception. Similarly, there is a good correlation between BFE and SE (Figure 5a), for all powder types, where an increase in BFE leads to an increase in the SE values.

Strondl et al. [5] observed similar results in EB-PBF powders where the BFE and SE values increased after reusing. Clayton et al. [33] also compared the BFE results for virgin and reused powders and concluded that processing significantly increases the BFE values for reused powders.

Both BFE and SE results suggest that the reused powders and blends would not flow as freely as G_0_. The energy to move the blade in the rheometer is increasing for the reused powders and blends mainly due to their deviation from an overall spherical shape (affected by the pre-heating, blasting and sieving procedures), which causes greater interparticle interactions during testing of the powder samples in the FT4 instrument. As explained by Clayton et al. [33], a higher SE indicates increased mechanical interlocking and friction between particles that may potentially lead to flow problems. An interesting observation is that the BFE and SE values for the G_3_ powder is lower than the G_2_ powder. The reason behind this behavior is not well understood, and therefore this powder type needs to be studied more extensively. However, the authors speculate that the blends have a much higher concentration of non-spherical particles when compared to their respective genesis powders. This may be due to the fact that these powders were exposed to rolling/tumbling over a cumulative time of 48 h. This tumbling procedure may have caused increased physical deformation to the blended powders when compared to the individual genesis powders.

**Figure 6:**
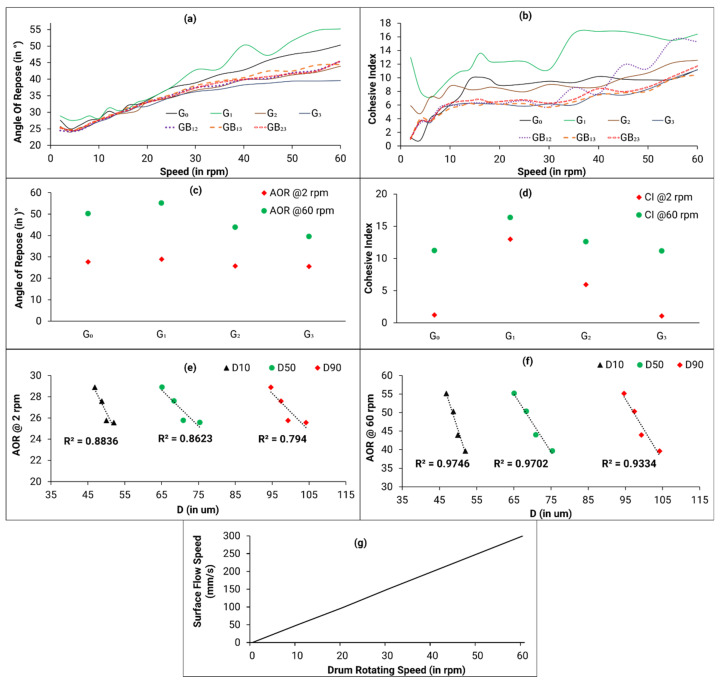
(**a**) AOR vs. speed of drum rotation for all powder types; (**b**) CI vs. speed of drum rotation for all powder types; (**c**) AOR at 2 and 60 rpm vs. number of reuse cycles; (**d**) CI at 2 and 60 rpm vs. number of reuse cycles; (**e**) AOR at 2 rpm vs. particle size (D values) for individual genesis powder types; (**f**) AOR at 60 rpm vs. particle size (D values) for individual genesis powder types; (**g**) relationship between drum rotating speed and surface flow speed (Redrawn from [34]). Note than G_0_ powder is considered an anomaly due to the fact that it has not undergone any processing, blasting, or sieving procedure.

Figure 6a presents a plot of the dynamic angle of repose vs. rotating drum speed for all powder types. It is observed that below 25 rpm, it is difficult to make a differentiation between the various powders and all powder have excellent flowability at 2 rpm. Above 25 rpm, the AOR decreases with an increase in number of reuse cycles. As an example, from Figure 6c, it can be observed that at the highest speed (i.e., 60 rpm), the AOR decreases from G_1_ to G_3_. It should be noted that G_0_ stands out, probably because this powder has not undergone any processing in the machine (pre-heating, blasting, sieving).

The AOR values at the lowest and highest drum rotation speed (i.e., 2 rpm and 60 rpm, respectively) were correlated with the particle size D_10_, D_50_, and D_90_ values. As can be seen from Figure 6e,f, an excellent correlation is observed where an increase in particle size leads to a decrease in the AOR value. It is recognised that larger particles tend to flow more easily than finer powder. It has been observed that the D_10_, D_50_, and D_90_ values increase with an increase in number of reuse cycles. Therefore, this increase in particle size is leading to a better flow in the rotating drum, thus suggesting that the flowability of the reused powders is better in this instrument.

Figure 6b presents the cohesive index (CI) at various drum rotation speeds for all powder types. The CI metric is linked to the fluctuations of the interface between the powder and air. The dynamic cohesive index of a powder depends on the magnitude of the cohesive forces between the particles. Therefore, a value closer to zero corresponds to a non-cohesive powder. Per the flow guidelines provided in the GranuDrum instrument software (Version 4.07, Granutools, Liège, Belgium), threshold values for cohesive index are: <5 very good, 5–10 good, 10–20 fair, 20–30 passable, 30–40 poor, >40 very poor. The CI follows the same trend (as observed in Figure 6b,d) as the AOR curves, where an increase in the number of reuse cycles leads to a decrease in the CI value (except G_0_).

The GranuDrum data interpretation guide mentions that the flowability of a powder is measured as a function of the shearing rates, and therefore rheological properties such as shear thinning or shear thickening could be evaluated with this instrument. If the powder AOR and CI increase with an increase in drum speed, the powder is said to show a shear-thickening behavior. All powders in this study show a shear-thickening behavior (Figure 6a,b). A powder material that shows a constant shear-thickening behavior is known to be a poor candidate for a dynamic process. That being said, there are portions in the CI vs. drum rotation speed curve (Figure 6b) where a somewhat constant cohesive index can be observed between speeds of 15 rpm and 40 rpm. The GranuDrum instrument (Granutools, Liège, Belgium) helps identify an optimum raking/re-coating speed. The relationship between the drum rotating speed and surface flow speed (i.e., raking speed in mm/s) is displayed in Figure 6g. It has been mentioned by the machine manufacturer [34,35], that when selecting raking speeds, one should look at areas that display a constant cohesive index.

As mentioned earlier, drum rotating speeds between 15 rpm and 40 rpm (corresponding to 75 mm/s and 175 mm/s, respectively, according to Figure 6g) show a constant cohesive index. Therefore, raking speeds between 75 mm/s and 175 mm/s should be considered when using powders for EB-PBF processes.

The effect of powder reuse on interparticle cohesion is contrary to the measurements done with FT4 instrument (higher BFE and SE with reuse). It is not clear at the moment whether the increase of the PSD with reuse is only associated with the agglomeration, the sieving, or a combination of both. Difference in flow regime and the sensitivity of the flowability to multiple powder modifications may explain the discrepancy in these results.

As mentioned by Brika et al. [16], powder flowability is not an inherent material property and is the ability of the powder to flow in a desired manner in a particular instrument. A powder may perform well in a certain instrument/piece of equipment while it may perform poorly in another. Thus, additional tests are required to better understand which regime better represents the behavior of the powder in an additive manufacturing (AM) machine.

**Figure 7:**
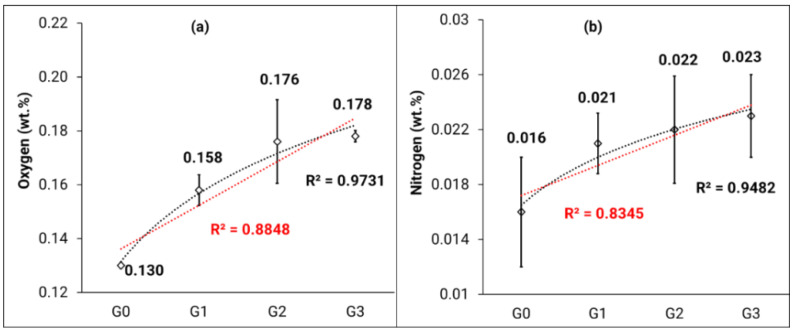
(**a**) Oxygen and (**b**) nitrogen concentration for G_0_, G_1_, G_2_, and G_3_ powder types along with linear (in red) and logarithmic (in black) trendlines. Error bars represent the standard deviation.

Figure 7a,b show that the average values for oxygen and nitrogen concentration increase with the number of reuse cycles. Specifically, a 37% and 44% increase was observed in the oxygen and nitrogen concentration, respectively, for G_3_ powder when compared to the G_0_ powder. However, the oxygen concentration remains lower than 0.18 wt.% and below the limit outlined by ASTM F2924-14 [11]. A logarithmic trendline seems to better fit the results when compared to the linear trendline (Figure 7a). From the logarithmic trendline, it can be deduced that the O_2_ concentration will exceed the 0.2 wt.% limit by 5–6 reuse cycles. The nitrogen concentration remains between 0.016 wt.% and 0.023 wt.% (Figure 7b), which is well below the 0.05 wt.% limit outlined by ASTM 2924-14 [11]. An increase of nitrogen concentration with the number of reuse cycles is observed; the logarithmic trend line suggests that this nitrogen pickup may reach a saturation level, suggesting that this is a surface contamination (formation of nitride or local concentration at the surface). The trend suggests that the maximum (i.e., 0.05 wt.%) will not be reached before a large number of reuse cycles.

Some other studies have also looked at the increase in oxygen and nitrogen content with an increase in number of reuse cycles. Ghods et al. [36] showed that the oxygen content was >0.20 wt.% after 11 reuse cycles. Grainger [29] observed a linear increase in oxygen and nitrogen concentrations in Grade 23 Ti-6Al-4V powder after reusing in the laser PBF process. However, after 30 builds, the oxygen concentration remained below 0.20 wt.%. These values are attributed to the fact that the starting oxygen concentration in Grade 23 Ti-6Al-4V is much lower than that of Grade 5 Ti-6Al-4V. Nandwana et al. [37] observed an increase in the oxygen concentration from 0.13 wt.% to 0.18 wt.%; however, their nitrogen concentration remained the same over five reuse cycles for a Ti-6Al-4V powder.

There are numerous factors which contribute to oxygen pickup in Ti-6Al-4V powders processed through EB-PBF. One of the possible reasons for the increase of oxygen pickup is the fact that the powder is exposed to the ambient atmosphere and moisture when transferred from the machine to the PRS for part recovery, and then transferred from the PRS to the sieve. A study performed by Vluttert [38] shows that Ti-6Al-4V can pick up about 0.2 wt. % moisture (relative to dry weight) when left in an environment that was set to 25 °C and 90% humidity. Therefore, the presence of water vapor in the air may have a reasonable impact on oxygen pickup. Montelione et al. [39] compare the EB-PBF process to gas tungsten arc welding and suggest that when the powder is exposed to air, the water molecules adsorbed on the particle surface may dissociate during heating, allowing the oxygen to diffuse in the metal alloy. In addition, the deformation caused by blasting and sieving can also lead to an acceleration of the oxidation due to creation of new oxidation prone surfaces. Shvab et al. [40] describe that this will lead to a passive oxide layer formation that may diffuse inward and reform during heating cycles. This oxide layer may also translate into the melt pool upon melting, and cause instabilities that form balling defects, thus hindering part consolidation [41]. Furthermore, oxygen pickup can also occur if the machine is not under vacuum, when the powder is stored in the hoppers, in between the builds. Finally, the major source of oxygen pickup is the reaction of titanium at high temperature with residual oxygen in the atmosphere of the machine. In addition, Mizuno et al. [42] noted that all oxides in titanium alloys dissolve above 400 °C and the oxygen diffuses into the bulk through the bulk diffusion process.

The EB-PBF process takes place at a relatively high temperature where the titanium can react with residual oxygen. Moreover, the high temperature can also result in oxygen diffusion as described by Attalla et al. [43] (build chamber ≈ 10^−4^ mbar and electron beam column ≈ 10^−7^ mbar). The oxygen and nitrogen can dissolve interstitially into the titanium lattice during solidification of the melt pool, and form oxides and nitrides [36]. Such oxides and nitrides are known to be detrimental to the fatigue performance of manufactured parts [44]. As stated by Donachie [45], an increase in the oxygen and nitrogen content in solution can lead to an increase in the strength and decrease in the ductility which further leads to embrittlement.

### 3.2. Evaluation of the Relative Performance of Powders

It is quite challenging to deploy a unified metric to capture the absolute suitability of powders for PBF AM processes. The powder metrics measured are highly different in nature and may impact the PBF process and final part properties in different ways. For instance, powder metrics variations may have opposite effects, i.e., some characteristics may lead to an improved behavior in the AM process, others may lead to a deterioration of the AM process behavior, while some powder metrics may have limited effect. In literature, this is poorly understood at the moment. Variability is still relatively high for some of the powder measurement techniques, and is influenced by the testing equipment type, the testing conditions, the powder storage and handling conditions, the operator skill, the calibration of instruments, and many other factors. Care must be exercised in interpreting each powder metric result individually. Although it is challenging to capture the absolute suitability of powders for PBF AM processes via a consolidated index, there is a potential to describe the relative change in powder properties with respect to a reference state, if such reference should exist. A reference state, for instance, is often considered to be the virgin powder. Two examples of calculations of relative changes from a reference state are presented below.

#### 3.2.1. Relative Performance Evaluated via Radar Diagram

**Figure 8:**
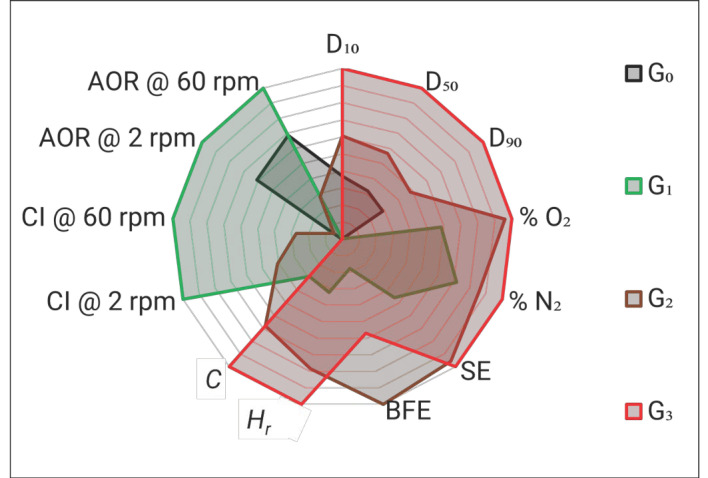
Radar diagram comparing the performance of G_0_, G_1_, G_2_, and G_3_ powders w.r.t the D values, SE, BFE, nitrogen (in wt.%), oxygen (in wt.%), *Hr*, *C*, CI, and AOR.

To understand the sensitivity of response to change of the various powder characteristics from a reference powder (G_0_ in this work), the various properties measured were normalized to a range of 0 to 1 and a radar diagram of the normalized indices for the G_0_, G_1_, G_2_, and G_3_ powders was plotted in Figure 8. The normalization was done to transform the data into a dimensionless data sequence for ease of comparability. For this study, the approach used for normalizing all metrics was taken from Mehat et al. [46]:

(1) $$x_{i}^{*}\left(k\right)=\frac{x_{i}^{\left(0\right)}\left(k\right)-\min\;x_{i}^{\left(0\right)}\left(k\right)\;}{\max\;x_{i}^{\left(0\right)}\left(k\right)-\min\;x_{i}^{\left(0\right)}\left(k\right)}$$

where $x_{i}^{\left(0\right)}\left(k\right)$ is the measurement of the quality characteristic, $\max\;x_{i}^{\left(0\right)}\left(k\right)$ is the largest value of $x_{i}^{\left(0\right)}\left(k\right)$, and $\min\;x_{i}^{\left(0\right)}\left(k\right)$ is the smallest value of $x_{i}^{\left(0\right)}\left(k\right)$.

In accordance with the powder suitability criteria created in Figure 8, the smaller the area covered by the radar diagram, the higher the powder suitability for the EB-PBF process. It is observed that the G_0_ powder has the smallest area, followed by G_1_, G_2_, and G_3,_ indicating that the powder is becoming less suitable with the increasing number of reuse cycles.

In this study, the authors evaluate the reused powders through various performance metrics such as morphology, size distribution, basic flow energy, specific energy, bulk density, tap density, Hausner ratio, Carr index, cohesive index, angle of repose, oxygen content, and nitrogen content. Each metric contributes additional knowledge and information regarding the powders, as per Table 1. However, performing all of these tests can be extremely time-consuming and expensive. Furthermore, acquiring results for all these metrics may not necessarily be relevant to a specific user or application. Therefore, it is up to the user to determine which tests are relevant for their own work based on the information that is provided for each characterization technique (Table 1 may be used for this purpose). For example, a user looking at manufacturing parts for aerospace applications will need to adhere by ASTM F2924-14, and therefore this user must evaluate the % O_2_ and % N_2._ Conversely, if a user is looking at manufacturing medical models solely for the purpose of education and training, then they may require good dimensional accuracy and surface finish for their model. Therefore, they must evaluate the particle size distribution (D values) and flowability metrics (e.g., BFE and SE) as these affect the layer homogeneity, proper spreading upon raking, minimum feature size, and surface finish as described in Table 1.

#### 3.2.2. Relative Performance Evaluated via Performance Index

Efforts in assessing powder suitability for powder bed fusion were undertaken by Brika et al. [16]; they differentiated powders and assessed three powder types by calculating a figure of merit called AMS (additive manufacturing suitability). While in this present study, the authors are not assessing an absolute suitability criterion due to challenges aforementioned; this approach is deployed herein to assess if this index can be used to quantify the relative changes in powder properties with respect to a reference state (G_0_ in this work). Since the current study focuses on EB-PBF powder suitability, the index will be referred as “ESF” (electron beam powder bed fusion suitability factor) and is inspired from Brika et al. [16]. As such, for each powder reuse cycle, a cumulative sum of the normalized index presented in Figure 8 will provide an objective function:

(2) $$\mathrm{ESF}=\frac{\left(D_{10}+D_{50}+D_{90}+O_{2}+N_{2}+\mathrm{SE}+\mathrm{BFE}+H_{r}+C+\mathrm{CI}\;@\;2\;\mathrm{rpm}+\mathrm{CI}\;@\;60\;\mathrm{rpm}+\mathrm{AOR}\;@\;2\;\mathrm{rpm}+\mathrm{AOR}\;@\;60\;\mathrm{rpm}\right)}{13}$$

The ESF values for G_0_, G_1_, G_2_, and G_3_ powders are 0.176, 0.504, 0.609, and 0.659, respectively. It is assumed that the lower deviation from the ESF value with respect to G_0_, the more the properties of the powder will depart from the reference state. This is another example of efforts towards expressing the relative change in powder characteristics.

As a conclusion, this study looked into comparing the influence of multiple reuse cycles, as well as powder blends created from reused powder, through various performance metrics such as morphology, size distribution, basic flow energy, specific energy, bulk density and tap density after 500 taps, Hausner ratio and Carr index, cohesive index, angle of repose, oxygen content, and nitrogen content. In accordance with the authors’ hypotheses established in Shanbhag and Vlasea [23], it was observed that reusing modifies the powder significantly when assessing the various performance metrics. While parts were printed during this study, no significant differences were observed during the different build cycles or general appearance of the printed components. The evaluation of the reuse of the powder on the microstructure and properties of the parts was beyond the scope of this study. Future research efforts will rely on this present study and will look into assessing the microstructure and mechanical properties of the parts to be able to understand the impact of reusing powders on part performance.

## 4. Conclusions

Investigation into the effect of plasma-atomized Grade 5 Ti-6Al-4V powder reuse on the powder properties as well as properties of powder blends led to the following conclusions:

(1) The SEM micrographs of the various powder types show extensive physical modification to the surface of the particles, with increasing degree of powder reuse. The micrographs depict features such as elongated particles, broken particles, clip-clap, deformed particles, particle with molten specks, and agglomerates. The broke, shattered, clip-clap, and deformed particles are attributed to the powder recovery process (i.e., blasting, sieving) and tumbling process; the particles with molten specks, elongated particles, and agglomerated particles are attributed to the high temperature conditions leading to overheating and smelting of particles and satellites.
(2) The D_10_, D_50_, and D_90_ values increase with increasing degree of powder reuse. Specifically, a 7%, 10%, and 7% increase was observed in the D_10_, D_50_, D_90_ values, respectively, for G_3_ powder when compared to the G_0_ powder. This observation is attributed to the agglomeration of powder particles.
(3) The *ρ*_0_ remained unchanged for all powders, however, the *ρ*_500_ increases with increasing degree of powder reuse. Specifically, a 3% increase was observed in the *ρ*_500_ value for G_3_ powder when compared to the G_0_ powder. The $H_{r}$ and C values show an increase with an increase in number of reuse cycles. Specifically, a 3% and 30% increase was observed in the $H_{r}$ and C values, respectively, for G_3_ powder when compared to the G_0_ powder. This trend indicates modification of reused powders from their virgin state. These observations have been attributed to the deviations from spherical morphology, for the reused powder, as observed in the SEM micrographs. Due to these deviations, uneven raking and non-homogenous layers may be obtained.
(4) The BFE and SE values increase with increasing degree of powder reuse. This suggests that the reused powder is more cohesive than the virgin powder. Specifically, an 18% and 15%, increase was observed in the BFE and SE values, respectively, for G_3_ powder when compared to the G_0_ powder. This behavior is attributed to the mechanical interlocking and friction between particles (caused by the non-spherical morphology).
(5) The dynamic AOR and CI values decrease with an increase in the number of reuse cycles. This behavior is attributed to the agglomeration and increase of the D_10_, D_50_, and D_90_ with the number of reused cycles. The variations of the metrics with drum rotating speed may help identify a range of optimum raking speeds that can be used for these powders when being used in the EB-PBF machine.
(6) The O_2_ and N_2_ concentration remain below the limits outlined by ASTM F2924. However, a gradual increase has been observed with increasing degree of powder reuse. Specifically, a 37% and 44% increase was observed in the O_2_ and N_2_ concentration, respectively, for G_3_ powder when compared to the G_0_ powder. From the logarithmic trendline, it can be deduced that the O_2_ concentration will exceed the 0.2 wt.% limit by 5–6 reuse cycles.
(7) A unified powder quality score or powder quality metric was established to compare the effect of powder reuse on the various powder performance metrics.

## Figures and Tables

**Table 1:** Powder suitability criteria for use in the EB-PBF process.

Powder Characteristic	Requirements	What Other Characteristics does This Property Have an Influence on	Performance Metrics That can Help Assess The Powder Property	Should This Performance Metric be Maximized (↑), Minimized (↓) or Kept Constant (↔)
Morphology	Spherical and equiaxed to increase flowability and avoid interparticle friction and mechanical interlocking	Powder bed packing density and the final component density, surface finish	Sphericity	↑
Apparent density	Should be high for improved heat conduction in the EB-PBF process	Heat conduction, sample swelling and overheating	*ρ*_0_	↑
Compressibility	High compressibility is desirable to be able to achieve higher packing density in the powder bed	Powder bed packing density, layer thickness, heat conduction	Carr index (C)	↓
Particle Size distribution (PSD)	Stay within the manufacturer’s size range and not drastically increase in order to obtain small feature sizes and thinner powder layers	Minimum layer thickness, minimum achievable feature size, build quality, surface finish	D_10_, D_50_ and D_90_ values	↔
Chemical composition	The oxygen and nitrogen concentration should stay within allowable concentration limits	Mechanical properties such as toughness, ductility of final parts, embrittlement	Oxygen (in wt.%) and nitrogen (in wt.%)	↓
Spreadability	A dynamic cohesive index closer to zero corresponds to a non-cohesive powder. A cohesive powder leads to a sporadic or intermittent flow while a non-cohesive powder leads to a regular flow	Powder bed packing density, powder layer uniformity, rake-ability, easy flow in the hoppers (i.e., powder storage). Decreased spreadability and flowability may contribute to more defects in the final part	Cohesive index (CI)	↓
Flowability	Highly flowable powder minimizes the risk of mechanical interlocking and friction between particles and allows for smooth operation while raking and for uniform and homogenous distribution over the build plate		$\mathrm{Hausner}\;\mathrm{ratio}\;(H_{r}$)	↓
			Basic flow energy (BFE)	↓
			Specific energy (SE)	↓
			Angle of repose (AOR)	↓

**Table 2:** Nomenclature and description of the various powder types investigated for the current study.

Powder Type	Description
G_0_	Virgin powder
G_1_	Powder that was used 1 time
G_2_	Powder that was used 2 times
G_3_	Powder that was used 3 times
GB_12_	50% G_1_ + 50% G_2_
GB_13_	50% G_1_ + 50% G_3_
GB_23_	50% G_2_ + 50% G_3_

## Data Availability

The data presented in this study are available on request from the corresponding author.
